# Breakfast Quality and Insulin Resistance in Spanish Schoolchildren: A Cross-Sectional Study

**DOI:** 10.3390/ijerph20021181

**Published:** 2023-01-09

**Authors:** María Dolores Salas-González, María del Carmen Lozano-Estevan, Aránzazu Aparicio, Laura M. Bermejo, Viviana Loria-Kohen, Rosa M. Ortega, Ana M. López-Sobaler

**Affiliations:** 1 VALORNUT Research Group, Department of Nutrition and Food Science, Faculty of Pharmacy, Complutense University of Madrid, 28040 Madrid, Spain; 2 IdISSC, 28040 Madrid, Spain

**Keywords:** breakfast, breakfast quality, children, insulin resistance

## Abstract

Background: Breakfast has traditionally been considered one of the most important meals of the day; however, there is little evidence for the influence of breakfast quality and insulin resistance (IR). This study aimed to assess the quality of breakfast in a group of schoolchildren, and its association with IR. Methods: A cross-sectional study with 852 children (8–13 years) was carried out. Fasting plasma glucose, insulin and anthropometric parameters were measured. A three-day dietary record was used to assess their diet and to calculate the Breakfast Quality Index (BQI). The sample was divided into tertiles according to the BQI (tertile 3: better breakfast quality). The homeostatic model was used to assess insulin resistance (HOMA-IR), and IR was defined as HOMA-IR > 3.16. Results: The prevalence of IR was 5.2%. The mean BQI score was 4.50 ± 1.25, and boys had lower scores than girls. Children in the BQI tertile 3 had a better global diet quality. In boys, being in the BQI tertile 3 was associated with a lower risk of IR (OR [95% CI]: 0.10 [0.01–0.77], *p* < 0.05). Conclusions: A higher-quality breakfast was associated with better overall diet quality and a lower risk of IR, especially in boys.

## 1. Introduction

In the countries of the Mediterranean basin, breakfast has traditionally been considered one of the most important meals of the day [1]. Despite this, European representative national surveys indicate that skipping breakfast is a common trend [2,3]. In particular, the frequency of breakfast skipping increases in adolescents and is higher in girls [4]. In addition, a high percentage of children follow a low-quality breakfast, considering the calories provided or the foods included in this meal [5,6].

The regular consumption of a good-quality breakfast is associated with various benefits in children and adolescents, such as adherence to higher overall quality diets [7,8], lower body mass index (BMI) [9,10], better cognitive performance [11], and higher levels of well-being and quality of life [12,13]. On the other hand, breakfast skipping predicts increases in weight during the transition from adolescence to adulthood [4,14]. Given that childhood represents a critical period of human development in which changes in health behaviours can be maintained into adulthood [15], it is important to monitor the acquisition of healthy dietary and lifestyle habits from the earliest years of life.

With the growing obesity epidemic, the incidence of insulin resistance (IR) in childhood and adolescence has increased worldwide [16]. In addition, IR is associated with other components of the metabolic syndrome; it is one of the key components in the development of type 2 diabetes mellitus [17], and it is associated with other cardiometabolic diseases [18]. 

Most studies examining the relationship between breakfast and IR have focused on the effect of skipping breakfast in both children [4,19,20,21,22,23] and adults [24,25,26]. However, some researchers point out that it is also important to analyse the quality of breakfast and not simply whether or not breakfast is eaten [27,28]. There is a difference between promoting just having breakfast and promoting having a quality breakfast, as just having breakfast may promote the increased consumption of sugary cereals and other nutrient-poor foods [29,30]. 

A quality and sufficient breakfast can contribute to appetite regulation. Eating breakfast leads to acute improvements in appetite, satiety and glycaemic control compared to skipping breakfast [31,32]. However, these responses are stronger when breakfast contains a higher amount of protein than when it is lower in protein, as it induces beneficial changes in hormonal (reduced ghrelin and increased peptide YY concentrations) and neural signals related to appetite [33]. These effects are independent of the type of breakfast usually eaten [34]. A breakfast rich in fiber (with fruits and cereals) can improve glycaemic control and possibly prevent sugar crashes between meals [35]. In addition, fiber is susceptible to colonic fermentation, which may result in the production of short-chain fatty acids that, upon entering the circulation, may attenuate hepatic glucose production and stimulate glucagon-like peptide 1 secretion; these effects may modulate insulin sensitivity [35]. On the other hand, the slow absorption and digestion of starch at one meal (such as breakfast) may improve carbohydrate tolerance at the next meal [35]. All these aspects are important because hyperglycemia can reduce nitric oxide availability and increase glycation end products and oxidative stress which are related to endothelial damage and atherogenesis [36]. In addition, hyperglycemia can increase gut permeability and increase liver and adipose tissue inflammation by increasing bacterial lipopolysaccharide in system circulation, which can produce insulin resistance [37].

Although there is no universal definition of an ideal breakfast due to geographical and cultural variability [38], different criteria have been proposed to assess the quality of breakfast, based primarily on the foods that form part of the meal [39,40,41]. One of the proposed indices is the Breakfast Quality Index (BQI), which takes into account the characteristics of the Mediterranean diet [42] and was proposed using a dataset of 4332 children and adolescents aged 8–17 years in Spain, with 6.5% of whom did not eat breakfast. Among those who did eat breakfast, higher BQI scores were associated with improvements in the ratios of macronutrients to energy intake and with improvements in Ca and MUFA: SFA intake.

The BQI has subsequently shown associations between breakfast quality and overall diet quality in other populations. In a representative sample of Spanish children and adolescents, the BQI was associated with higher diet quality as assessed by the Nutrient Rich Foods Index (NRF9.3) and higher intakes of micronutrients (vitamins A, D, C, B1, B2, B6, niacin, folate, calcium, potassium, and magnesium) [7]. In Brazilian adults, those with a high BQI had a higher intake of fruits and vegetables and a higher breakfast and daily intake of energy, carbohydrates, fibre, total sugar, sodium, potassium, phosphorus, thiamine, riboflavin, niacin, folate and vitamins B6, A, C and D, and fewer trans fatty acids, compared to those with a lower BQI [43].

Finally, higher breakfast quality as measured by the BQI has been associated with lower prevalence of obesity and cardiometabolic risk [27,44], and lower caries frequency [45].

Current research supports the association between skipping breakfast and IR. However, although breakfast quality has been associated with other diseases at school age, there are few studies linking breakfast quality and IR in this age group. Therefore, the aim of our study was to assess breakfast quality in a group of children and to analyse its relationship with IR.

## 2. Materials and Methods

### 2.1. Study Sample

**Figure 1:**
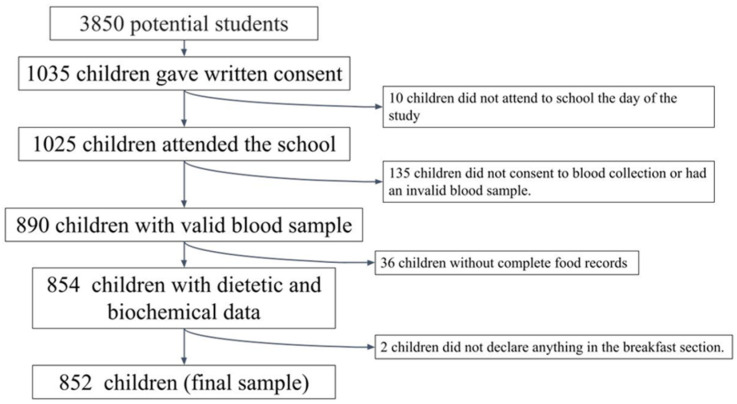
Flowchart of the selection process.

A cross-sectional observational study was conducted, including a convenience sample of schoolchildren aged 8 to 13 years from 5 Spanish provinces (A Coruña, Barcelona, Madrid, Seville, and Valencia). In each province, schools were randomly selected from a list of primary schools, with at least two classes per grade. The management team of 55 schools was contacted by phone and an interview was arranged to explain the characteristics and objectives of the study. After approval by the management team (22 schools), the families of children of the target age were called for a meeting to explain the purpose and details of the study, and to clarify any doubts. Subsequently, the students’ parents or legal guardians were then asked to sign a written consent for their children to participate in the study. Similar samples have been used in other studies [46,47]. Figure 1 shows a flowchart of the selection process.

The inclusion criteria to participate in the study were being between 8 and 13 years; studying in the fourth, fifth or sixth grades of primary school; having signed informed consent from the child’s parents and/or guardians; and the acceptance of all study conditions.

The exclusion criteria were the presence of any disease that could modify the results of the study, such as metabolic or chronic diseases (diabetes, renal pathologies, etc.); the impossibility of coming to the centre on the agreed days to carry out the tests; a pharmacological treatment that could interfere with the results of the study; and not declaring the consumption of any food at breakfast.

The participating children underwent a socio-demographic, physical activity, dietary, anthropometric and biochemical study at their school, which was managed by qualified research staff. Measurements were made between February 2005 and June 2009. 

This study was conducted in accordance with the guidelines established in the Declaration of Helsinki, and the Human Research Review Committee of the Faculty of Pharmacy of the Complutense University of Madrid approved all procedures involved in this study.

### 2.2. Socio-Demographic Data

A self-completed survey by the parents/guardians of the children was used, including questions related to the demographic characteristics of the children (sex, date of birth, and maximum level of education attained by the parents).

### 2.3. Anthropometric Data

Anthropometric survey measurements were carried out by qualified personnel. The study was carried out in the schools themselves in the morning and following the standards established by the World Health Organization (WHO) [48]. For all measurements, the subjects were barefoot and wore only underwear. 

Body weight and height were determined using a digital electronic scale (range 0.1–150 kg, accuracy 100 g; Seca Alpha, GmbH, Igny, France) and a Harpenden digital stadiometer (range 70–205 cm, accuracy 1 mm; Pfifter, Carlstadt, NJ, USA). The BMIs of the subjects were calculated using weight (kg)/height^2^(m^2^). Subsequently, the BMI z-score (zBMI) was calculated according to the WHO growth standards [49]. Cut-off points were set according to the standard deviation of the WHO z-score (z < −2: underweight, z: −2–1: normal weight, z:1–2: overweight, and z > 2: obese) [50].

The tricipital skinfold was measured on the right side of the body using a Holtain skinfold caliper (constant pressure of 10 g/mm^2^ (range: 0–39 mm) and 0.1 mm accuracy, Holtain Ltd., Crymych, Wales) following the recommendations by the ISAK [51]. Fat mass (FM) was obtained using the formula proposed by Dezemberg et al. in 1999 [52].

### 2.4. Physical Activity Data

The parents completed an adapted questionnaire on their children’s daily physical activity [53], which had been used previously in other studies [46,47,54,55]. The questionnaire asked about the time spent on different activities during the day: sleeping; being in class; study time; time spent at different meals; time spent in sedentary play (with the PC, video game consoles or watching TV); time spent playing actively on the street and play time at home; time spent in the gym or doing sports activities at school, at recess and during after-school activities; time and mode of travel between home and school; and other activities.

The time spent on the different activities was then grouped into four categories: sleep, very light activities (activities performed lying down, sitting or standing—painting, playing an instrument, cooking, etc.), light activities (equivalent to walking on a flat surface at 4–5 km/h, cleaning the house, golf, table tennis, etc.), and moderate and/or vigorous activities (physical activities requiring a higher energy expenditure, such as cycling, skiing, tennis, dancing, basketball, football, rugby, and running).

An activity coefficient was established for each subject by multiplying the time spent on each activity by the established coefficients [56,57] (1 for sleep and rest activities, 1.5 for very light activities, 2.5 for light activities, 5 for moderate activities, and 7 for vigorous activities), and then dividing by 24 h. Once the individual physical activity coefficient was obtained, it was used to establish the physical activity category for the calculation of energy expenditure (EE) according to the Institute of Medicine (IoM) equations [58].

### 2.5. Dietary Data

For dietary intake, a “food record” was conducted for three consecutive days, including a weekend day (Sunday to Tuesday) [59]. The parents were asked to record the weight, if possible, or to use estimated measurements at home of all of the foods consumed by their children outside of school. On Monday and Tuesday, the research staff visited the school canteen, recorded the foods on the menu, and weighed the amounts of foods served to each of the children who stayed for lunch in the canteen, and what each child left on his or her plate. The DIAL software (Alce Ingeniería, Madrid, Spain) was used to process the dietary surveys [60]. This software includes a database with coded foods and another with standard recipes, which was completed with the specific recipes of the menus served in the school canteens. In this way, the software disaggregates the recipes into their ingredients. In addition, the program includes another database with weights and household measures or commercial units (spoon, cup, pinch, handful, drop, bottle, can, slice, etc.). This makes it possible to record the consumption of each food or recipe in grams, in c measures, or in consumption units. Finally, this software also includes a database with the food composition tables proposed by Ortega et al., 2021 [61]. The program calculates the intake in grams and servings of the different food groups and also the energy and nutrient intake, both for the total and for each of the meals (breakfast, lunch, snack, dinner, etc.).

The following dietary parameters were calculated for the total diet and breakfast: energy; macronutrients (protein, lipids and carbohydrates); monounsaturated (MUFA), saturated (SFA) and polyunsaturated (PUFA) fatty acids; simple sugars (mono- and disaccharides); free sugars; fibre; and calcium. The amounts of fruit, natural juices, dairy, cereals and derivatives, olive oil, butter and margarine consumed at breakfast were also calculated. 

Energy expenditure (EE) was calculated according to the IOM equations [58]. The possible underestimation or overestimation of energy intake was determined as the discrepancy between energy intake (EI) and EE, as measured by the formula (EE-EI) × 100/EE. This formula provides a percentage of possible underestimation (if the values are positive) or overestimation of intake (when the values are negative) [62].

The Healthy Eating Index (HEI-2015) was used to assess diet quality. It is composed of 13 components: 9 for adequacy and 4 for moderation. The items of whole grains, dairy and fatty acid ratios were scored from 0 to 10; the items of total fruits, whole fruits, vegetables, seafood and vegetable proteins, green vegetables, legumes and protein foods were scored from 0 to 5; and the items of moderation including refined grains, sodium, free sugars and saturated fats were scored from 0 to 10 [63].

### 2.6. Definition of Breakfast

The definition of breakfast is that it is the first meal of the day that breaks the fast after the longest period of sleep; it is consumed within a couple of hours (i.e., usually 2–3 h) after waking up, comprises food and beverages from at least one food group, and it can be consumed anywhere [44]. In our study, breakfast was defined as the first meal of the day by the participants. The number of days when the participants had breakfast was recorded, and those who did not eat breakfast on any of the dietary study days were excluded. 

### 2.7. Breakfast Quality Index (BQI) 

The Breakfast Quality Index (BQI) designed by Monteagudo et al. [42] to evaluate the quality of breakfast in children and adolescents in the Mediterranean area was calculated. This index is composed of 10 items assessing the food groups (4 items) and the energy and nutrients (6 items) of public health interest. The food groups included in the original BQI [35] are (I) fruit (whole fruit, tomato or fresh juice), (II) dairy products (whole or skimmed milk, cheese or yoghurt), and (III) cereals and cereal products (where only unsweetened cereals or bread are included). These items score 1 point if they are consumed at breakfast on at least one of the three days of the food record. In addition, a fourth item is considered if cereals, fruits and dairy products are included in the same breakfast on at least one of the days studied. The energy and nutrient items included in the BQI are (I) “include olive oil”, (II) “do not include butter or margarine”, (III) “provide 200–300 mg of calcium”, (IV) “provide <5% of total daily energy from simple sugars”, (V) “have a ratio of MUFA to SFA (MUFA:SFA) > medium”, and (VI) “provide 20–25% of total daily energy intake at breakfast”.

In our study, we applied the BQI with two modifications. First, we adopted the modification proposed by Arenanza et al. [27], scoring with 1 point the item of “having a ratio of monounsaturated to saturated fatty acids (MUFA:SFA) ≥ 2:1”. Secondly, and considering the age range of our sample and the fact that the dietary reference values are different for each age, we adopted the criterion already used by other authors [7,43], which scores calcium items positively when the total exceeds 20% of the population reference intakes (PRI) proposed by the EFSA [64].

Attending to the final items included, the BQI scores ranged from 0 to 10 (a higher BQI score carried a higher breakfast quality).

### 2.8. Biochemical Data

Blood samples were drawn via venipuncture between 08:00 and 09:00 h after 12 h of fasting. The nursing staff verified the adequacy of the fasting period prior to blood collection. The samples were collected at the centre. 

Plasma glucose was determined colorimetrically using the glucose oxidase-peroxidase method [65] (Vitros GLU Slides, Rochester, NY, USA; CV = 2–8%). Fasting insulin was measured via an immunochemiluminometric assay [66] (Abbott Diagnostics, Madrid, Spain; CV = 4.8%). The homeostasis model assessment value (HOMA-IR) was used to reflect the degree of IR [67,68]: HOMA-IR = [basal glucose (mmol/L) × basal insulin (μU/mL)]/22.5. IR was considered to be present when the HOMA-IR was greater than 3.16 [69].

### 2.9. Statistical Analysis

Descriptive data were expressed as means and standard deviations. For a comparison of the means, the Mann–Whitney U test was used if the distribution of the variables was not normal, and the Student’s t-test was used for normal distributions along with the two-way ANOVA test. Categorical variables were compared using the Chi2 test and the Z-test of proportions. The population was divided using BQI tertiles for analysis (Tertile 1 (T1): <4 points, Tertile 2 (T2): 4 points, and Tertile 3 (T3): >4 points). Logistic regression analysis was performed to identify the aspects of breakfast associated with IR, and odds ratios (OR) and 95% confidence intervals (CI) were reported. For dairy consumption, given that almost all children included dairy in their breakfast, the risk of including at least half a portion of dairy against including less dairy was analysed. The risk of compliance or non-compliance with the remaining items was analysed for the remaining items. The consumption of whole fruit and natural juice, as well as the inclusion of butter and margarine, was also analysed separately. A *p*-value of <0.05 was considered as being statistically significant. All calculations were performed with IBM SPSS Statistics for Windows, version 28.0 (Armonk, NY: IBM Corp, published in 2021).

## 3. Results

The potential initial sample size was approximately 3850 participants. Children in the same age range had been previously studied in another context [38,39]. The sample size was calculated by taking into account the number of schools that agreed to participate (n = 22), the number of pupils per classroom (n = 25), and the number of classrooms per grade (between two and three). Subsequently, 1035 schoolchildren (49.2% boys) obtained written consent from their parents or guardians to participate in the study. Ten participants did not attend school on the day of measurement and were therefore excluded from the analysis. Valid dietary data were obtained from 965 children (48.8% boys), and one boy and one girl skipped breakfast and were therefore excluded from the analysis. Of these children, valid blood samples were obtained from only 852 children (48.5% boys) who constituted the final sample of this study. 

Most of the children (98.0%) had breakfast on all three days, 1.3% had breakfast on two days, and 0.7% had breakfast on only one day. 

Table 1 shows that boys have a higher zBMI, higher fasting glucose values, and a higher percentage of obesity than girls, and more boys engage in more than 60 min of moderate or vigorous physical activity per day compared to girls. Girls have higher insulin, HOMA-IR values, and prevalence of IR than boys.

Children with IR are older (10.5 ± 1.0 vs. 10.1 ± 0.9 years, *p* < 0.05), have higher zBMI (1.46 ± 1.11 vs. 0.64 ± 1.12, *p* < 0.05), have a higher prevalence of obesity (29.5% vs. 11.4%, *p* < 0.05), and are less active than children without IR (activity coefficient: 1.49 ± 0.10 vs. 1.53 ± 0.11, *p* < 0.05). 

Table 2 shows the breakfast quality as measured by the BQI according to gender. Girls scored higher on the BQI. More girls than boys include cereals, fruits and juices in their breakfast. The most compliant items are the consumption of dairy products and calcium, followed by the consumption of cereals and cereal products. The items with the lowest compliance are the MUFA/SFA ratio, the consumption of all three food groups at breakfast, and the inclusion of olive oil.

Table S1 shows that the mean daily energy intake is 2105 ± 350 kcal/day, with an underestimation of 1.8%, and both the caloric intake and underestimation are higher in boys. Of this energy, breakfast contributes 17.7% of the total energy intake. Boys consume less fruit and juice than girls at breakfast. However, when whole fruit and juice consumption were analysed separately, it was observed that there are no significant differences in whole fruit consumption, but girls consume more juice than boys. Boys consume more SFA at breakfast than girls. In terms of the total dietary intake, boys consume more calcium and SFA than girls and have a poorer diet quality, as measured by the HEI-2015.

Table S2 shows the anthropometric, biochemical and dietary data for breakfast, and the daily data according to the BQI tertiles. There are no differences in anthropometric or biochemical data. At breakfast, children in the T3 BQI have a higher energy intake; consume more fruits and juices, dairy, cereals and olive oil; have higher intakes of proteins, MUFA, PUFA, fibre and calcium; and have lower intakes of margarine, simple sugars and free sugars than those in BQI T1 and T2. In reference to the total diet, the T3 BQI children have a higher diet quality as measured by the HEI-2015 scores, higher energy, protein, fibre and calcium, and lower intakes of free sugars. Children who include fruit or juice at breakfast consume more fibre, both at breakfast (2.8 ± 2.6 g/day vs. 1.5 ± 1.1 g/day, *p* < 0.05) and in the total diet (17.3 ± 4.9 g/day vs. 15.9 ± 4.6 g/day, *p* < 0.05) than those who do not consume fruit at breakfast. In addition, children who eat some cereals and derivates at breakfast consume more fibre, both at breakfast (2.0 ± 2.1 g/day vs. 1.7 ± 1.1 g/day, *p* < 0.05) and in the total diet (16.5 ± 4.7 g/day vs. 16.0 ± 4.9 g/day, *p* < 0.05) than children who do not. Children who include a dairy product in their breakfast have higher calcium intakes, both at breakfast (327.8 ± 98.9 mg/day vs. 71.6 ± 81.3 mg/day, *p* < 0.05) and in the total diet (971.5 ± 215.6 mg/day vs. 679.1 ± 301.8 mg/day, *p* < 0.05). Finally, children consuming less than 5% energy from simple sugars at breakfast also consume less free sugars, both at breakfast (1.6 ± 0.8% kcal/day vs. 3.1 ± 1.5% kcal/day, *p* < 0.05) and in the total diet (8.0 ± 3.6% kcal/day vs. 9.0 ± 3.6% kcal/day, *p* < 0.05, respectively).

Table 3 shows the quality of breakfast by gender and IR. Boys with IR score lower on the BQI than boys without IR. There are no differences in girls in terms of the overall breakfast quality score, although more girls with IR than their non-IR counterparts include fruit and juice at breakfast and meet the recommendations for calcium and simple sugars.

Table S3 shows food, energy and nutrient intakes at breakfast, and total daily intake according to sex and HOMA-IR. At breakfast, girls with IR have a higher intake of lipids, MUFA, PUFA and SFA, and they have a higher intake of fruit and juice than girls without IR. However, when splitting between juice and whole fruit, there is a significant difference in juice intake, but not in whole fruit intake. There are no significant differences in any of the variables in boys.

Table 4 shows crude and adjusted logistic regression models for the association of breakfast quality, and each BQI item compliance and IR. Boys with a better breakfast quality (T3 of the BQI) have a lower risk of IR than those with a poorer breakfast quality. Including fruit and/or juices at breakfast is associated with an increased risk of IR, both in the total sample and only in girls. However, in a subsequent analysis considering separately the inclusion of whole fruit or juices, no increased risk of having IR is observed when whole fruit is included at breakfast, but there is a risk when juices are included. In addition, including butter increases the risk of having IR in the total sample. However, the risk disappears when analysing boys and girls separately. In boys, that risk could not be calculated with respect to the MUFA/SFA ratio and the daily energy intake of 20–25% at breakfast, because no boys with IR scored on this item.

## 4. Discussion

In this study, it was observed that the consumption of breakfasts with a higher overall quality was associated with a lower risk of IR, while the inclusion of natural juices or butter was associated with a higher risk of IR. 

Insulin resistance in our sample was 5.2%, comparable to that found in other age-matched populations in Greece [70], but very different from that observed in other studies [22,71,72,73,74], as the variability between them is very large. According to a study in Mexico with children under 12 years of age, 17.1% of the subjects had IR [71]. Karatzi et al. reported a prevalence of IR in children in Greece of 28.7% [22]. Furthermore, a comparison of the figures with other studies is difficult because the prevalence of IR has often been analysed only in overweight and/or obese children, such as in a study in India, where the prevalence of IR was reported to be 22.2% [72]; in a study in children aged 6–13 years in Iran, where the prevalence of IR was 43.4% [73]; or in children aged 6–18 years in Mexico, where the prevalence of IR was 56.3% [74].

A comparison of our results with those of other studies is limited by differences in the definition of breakfast, and the different criteria for assessing breakfast quality. The aspects taken into account to define a healthy and quality breakfast are variable and include the regularity of this habit, the energy contribution of breakfast, and its nutritional quality [28]. Then, it is important to focus not only on not skipping breakfast, but on having a good breakfast. In this sense, one of the most commonly used criteria for defining a healthy breakfast is to include at least three main food groups [38].

In this study, the percentage of schoolchildren skipping breakfast was lower than in other studies. A recent systematic review (children aged 2–18 years) that included 39 studies in 33 countries concluded that most of the investigations reported that 10–30% of children and adolescents skipped breakfast (with a large variability from 0.7 to 74.7%) [4]. This may be due to the age of the children studied, as the literature suggests that skipping breakfast is common in children and adolescents and increases with age [75].

In the present study, the mean BQI score was 4.5 points, indicating that the quality of breakfast could be improved [42]. This score was similar to those of other studies on Spanish children that used the same tool [7,27]. Including dairy products at breakfast and meeting the calcium intake at this meal were the items with the highest adherences in our sample. On the other hand, the inclusion of olive oil at breakfast and the MUFA/SFA ratio of this meal were the items with the lowest adherence, which may affect the quality of the lipid profile of the total diet. In this sense, previous studies with a similar schoolchildren sample have found that the intake of SFA is high [47]. It is also important to note that approximately 80% of children in our study took less energy than was recommended at breakfast, which can lead to impaired satiety throughout the day. Finally, 70% of schoolchildren in our sample included butter or margarine at breakfast on some days, which is well above other Spanish studies where 3–4% and 27% are reported [7,27], respectively.

In the present study, those in the highest tercile of BQI had a better overall diet quality, as measured by the HEI-2015. This is consistent with other authors who report that a better breakfast quality is associated with a better diet quality [7,44,76]. In addition, children with better BQI scores had higher intakes of fibre and calcium, and lower intakes of free sugars in their total daily intakes. It is important to take these results into account since sugar is a critical nutrient to control in diet, especially in children. In addition, the intake of fibre and calcium intake are insufficient in the average diets of Spanish children, and a high percentage of schoolchildren do not meet the dietary recommendations for these nutrients [7].

A higher-quality breakfast was associated with a lower risk of HOMA-IR among the boys in our study. This finding is consistent with a study in Brazilian adults [44]; however, in a study in Spanish children, BQI is neither positively nor negatively associated with the HOMA-IR [27]. Other researchers have suggested that breakfast quality may be causally related to appetite control and glycaemic control [35]. Even a poorer breakfast quality in adolescence has been associated with increased metabolic syndrome and higher glycaemia in adulthood [77].

The association between breakfast quality and IR in children may be due to the implication that a good breakfast quality helps to maintain an adequate weight status and prevent comorbidities associated with excess weight. However, in our sample, children with a higher-quality breakfast did not have a lower zBMI, and other researchers have demonstrated this association between a higher-quality breakfast and a better weight status [28,75]. It may also be due to the consumption of different foods included in a quality breakfast, such as dairy products, cereals and fruit.

In our country, breakfast is a key time for dairy intake (approximately 98% of our sample included dairy at breakfast). In fact, those schoolchildren who included dairy at breakfast had a higher total dietary calcium intake. This is interesting as dairy intake in adults has been shown to have beneficial effects on IR and overall cardiovascular health [78]. Dairy products contain calcium, vitamin D, lipids and bile acids. The combination of calcium, fats and bile acids in the gastrointestinal system is known to inhibit fat reabsorption and to improve the ratio of HDL cholesterol to LDL cholesterol [79], thus improving dyslipidaemia, which is strongly associated with IR [80].

The consumption of fibre from wholegrain cereals or fruit helps to control blood glucose levels, improves insulin sensitivity, and reduces atherogenic risk factors [44]. It should be noted that although the BQI distinguishes between sweetened and unsweetened cereals, it does not distinguish between wholegrain and refined cereals. However, despite this, we observed that those with higher BQI scores had a higher intake of fibre at breakfast and in the total day, and those who included fruit or cereals at breakfast had a higher intake of fibre at breakfast and in the total diet. 

In our study, those taking less than 5% of simple sugars had fewer simple sugars and less free sugars at breakfast and in the total diet. A high intake of sugars causes an increased glycaemic load in the diet, leading to β-cell dysfunction, inflammation and increased IR [81]. 

In our results, juice consumption was associated with an increased risk of IR. It is important to differentiate between whole fruit and juice consumption. In addition, although the participants were asked to indicate whether the juices were natural or not in our dietary study, they might not differentiate well between natural fruit juices, commercial juices and fruit nectars (the latter may have added sugars). The relationship between juices and IR is contradictory: in two meta-analyses in adults, one concluded that fresh orange juice is associated with a significant reduction in HOMA-IR [82], and the other concluded that fruit juice significantly increases HOMA-IR values [83], but both stated that the literature on this topic is still limited and that more studies are needed. In another meta-analysis of randomised controlled trials, the HOMA-IR value was significantly reduced with the intake of natural orange juice (≥500 mL/day) in healthy young adults, but not in those with metabolic disorders [84]. Contradictory data between juice and HOMA-IR are also present in the relationship between juice and excess weight, with some studies showing an increased risk of obesity [85,86], while others reporting no effect [87,88] or even finding a lower body weight in regular fruit juice consumers [89,90]. 

These contradictory data may be due, on the one hand, to the fact that natural orange juice is a good source of vitamin C. Vitamin C may improve glycaemic control due to its ability to improve the fitness of the plasma membrane as a potent glucose transporter following an increase in glutathione levels. On the other hand, orange juice contains simple sugars and does not provide the fibre that is present in whole fruit [78]. The soluble fibre provided by whole fruit slows the rate of glucose absorption, which reduces the glycaemic index of breakfast and the insulin response required to eliminate ingested glucose [91].

A noteworthy aspect of our study is that we studied a relatively large sample of schoolchildren considered to be healthy and with no previous diagnosis of diabetes, in which 5% of cases with IR were identified. There is little literature on IR in children in this age range, and this is one of the first studies to relate IR in children with indicators of a healthy breakfast. The use of the 3-day dietary recording technique to collect dietary information is also a strength, as it allows information to be collected on the variety and types of foods consumed, and on the different meals eaten during the day. Moreover, it is one of the most suitable methods for assessing diet quality using the index that we had used. 

As for the limitations of our study, the sample studied is a convenience sample, so the conclusions of our study will need to be confirmed in future research. It should be noted that, being a large community-based study, Tanner’s stratification by a clinician was not possible, and information on the pubertal status of the participating children was not included. As only the tricipital skinfold could be obtained in most participants, the method for estimating fat mass has limitations and is not the best method. Although the physical activity questionnaire has been used in other studies, it is not a validated questionnaire. Additionally, the errors in our dietary survey must be considered as these are self-reported data, which may be associated with a certain degree of underreporting. Finally, although in our study, the BQI describes breakfast quality in schoolchildren, and this is associated with better overall diet quality and lower IR, it is an index that has not been validated. In view of our results and those of other studies that have applied the same index [7,27], it needs to be validated in order to corroborate our results and to analyze the relationship between breakfast quality and health outcomes.

## 5. Conclusions

In conclusion, it is not enough for children to eat breakfast. However, a quality breakfast including dairy products, cereals (especially wholegrain) and whole fruit, and excluding foods that are rich in simple sugars and butter, contributes to improving the overall diet quality, and it is also associated with a lower risk of IR in school-age children, especially in boys.

## Figures and Tables

**Table 1:** Anthropometric, biochemical, socio-demographic and physical activity according to sex.

	Total (n = 852)	Girls (n = 440)	Boys (n = 412)
**Father’s highest level of education [% (n)]**			
No schooling or primary education	24.6 (210)	23.2 (102)	26.2 (108)
Secondary Education	34.6 (295)	34.8 (153)	34.5 (142)
University studies	29.7 (253)	28.0 (123)	31.6 (130)
Not determined	11.0 (94)	**14.1 (62)**	7.8 (32) *
**Mother’s highest level of education [% (n)]**			
No schooling or primary education	20.9 (178)	21.1 (93)	20.6 (85)
Secondary Education	39.6 (337)	38.4 (169)	40.8 (168)
University studies	33.1 (282)	32.5 (143)	33.7 (139)
Not determined	6.5 (55)	8.0 (35)	4.9 (20)
**Age and anthropometric data-X ± SD**			
Age (years)	10.1 ± 0.9	10.1 ± 0.9	10.1 ± 1.0
Weight (kg)	39.4 ± 9.2	39.6 ± 9.2	39.1 ± 9.3
Height (m)	143.4 ± 8.6	**144.1 ± 9.1**	142.7 ± 8.0 *
Body fat (%) #	27.6 ± 5.7	**29.3 ± 4.7**	25.8 ± 6.1 *
BMI (kg/m^2^)	19.0 ± 3.1	18.9 ± 3.0	19.0 ± 3.3
zBMI #	0.68 ± 1.13	**0.57 ± 1.04**	0.80 ± 1.20 *
**Nutritional status by BMI [% (n)]**			
Underweight	0.8 (7)	0.7 (3)	1.0 (4)
Normal weight	59.4 (506)	**63.2 (278)**	55.3 (228) *
Overweight	27.5 (234)	29.1 (128)	25.7 (106)
Obesity	12.3 (105)	**7.0 (31)**	18.0 (74) *
**Physical Activity**			
Activity coefficient [X ± SD]	1.53 ± 0.11	1.52 ± 0.11	1.53 ± 0.11
MVPA ≥1 h/day [% (n)]	31.8 (262)	**23.2 (98)**	40.9 (164) *
**Biochemical data-X ± SD**			
Glucose (mg/dL)	84.4 ± 9.7	**83.4 ± 10.0**	85.5 ± 9.2 *
Insulin (μU/mL)	6.3 ± 4.4	**7.1 ± 4.8**	5.5 ± 3.6 *
HOMA-IR	1.33 ± 0.97	**1.48 ± 1.08**	1.17 ± 0.80 *
Insulin resistance [% (n)]	5.2 (44)	**7.0 (31)**	3.2 (13) *

X: mean; SD: standard deviation; BMI: body mass index. MVPA: moderate and/or vigorous physical activity. Most variables follow a non-parametric distribution; variables with normal distribution are marked (#). For a comparison of means, the Mann–Whitney U test is used if the distribution of the variables is not normal, and Student’s t-test is used for normal distributions. Associations between categorical variables are analysed with the χ² test and the Z-test of proportions. Differences between the sexes are indicated by asterisks and bold type ( * *p* < 0.05).

**Table 2:** Breakfast Quality Index (BQI) and number of children complying with the index criteria by sex.

	Total (n = 852)	Girls (n = 440)	Boys (n = 412)
BQI (score) (0–10), X ± SD	4.50 ± 1.25	**4.58 ± 1.25**	4.41 ± 1.25 *
**Item BQI [% (n)]**			
Fruits and/or juice consumption	29.6 (252)	**33.6 (148)**	25.2 (104) *
Dairy products consumption	97.8 (833)	98.0 (431)	97.6 (402)
Cereals and derivate consumption	61.3 (522)	**65.2 (287)**	57.0 (235) *
Fruits, cereals, and dairy product in the breakfast	14.8 (126)	**18.0 (79)**	11.4 (47) *
Include olive oil	16.2 (138)	16.4 (72)	16.0 (66)
Absence of butter or margarine	29.3 (250)	30.5 (134)	28.2 (116)
Calcium (>20% PRI)	91.3 (778)	90.7 (399)	92.0 (379)
Simple sugar (<5% kcal/day)	46.6 (397)	43.4 (191)	50.0 (206)
MUFA/SFA ratio (≥2:1)	0.47 (4)	0.45 (2)	0.49 (2)
Energy intake from breakfast (20–25% kcal/day)	21.0 (179)	22.3 (98)	19.7 (81)

BQI: Breakfast Quality Index; X: mean; SD: standard deviation; PRI: population reference intakes; MUFA: monounsaturated fatty acids; SFA: saturated fatty acids. For comparison of the BQI, the Mann–Whitney U test is used. For comparison of the items, the χ² test is used. Differences between sexes are indicated by asterisks and bold type ( * *p* < 0.05).

**Table 3:** Breakfast Quality Index (BQI) and number of children complying with the index criteria, by sex and HOMA-IR.

	Total		Girls		Boys	
	HOMA-IR ≤3.16 (n = 808)	HOMA-IR >3.16 (n = 44)	HOMA-IR ≤3.16 (n = 409)	HOMA-IR >3.16 (n = 31)	HOMA-IR ≤3.16 (n = 399)	HOMA-IR >3.16 (n = 13)
BQI (score) (0–10), X ± SD	4.50 ± 1.24	4.50 ± 1.34	4.56 ± 1.24	4.71 ± 1.30	**4.43 ± 1.24**	4.00 ± 1.35 *
**Item BQI [*%* (n)]**						
Fruits and/or juice consumption	**28.8 (233)**	43.2 (19) *	**32.3 (132)**	51.61 (16) *	25.3 (101)	23.1 (3)
Dairy products consumption	97.7 (789)	100.0 (44)	97.8 (400)	100.0 (31)	97.5 (389)	100.0 (13)
Cereals and derivate consumption	61.0 (493)	65.9 (29)	65.3 (267)	64.5 (20)	56.6 (226)	69.2 (9)
Fruits, cereals, and dairy product in the breakfast	14.4 (116)	22.7 (10)	17.1 (70)	29.0 (9)	11.5 (46)	7.7 (1)
Include olive oil	16.0 (129)	20.5 (9)	15.9 (65)	22.6 (7)	16.0 (64)	15.4 (2)
Absence of butter or margarine	**28.6 (231)**	43.2 (19) *	29.6 (121)	41.9 (13)	27.6 (110)	46.2 (6)
Calcium (>20% PRI)	91.7 (741)	84.1 (37)	**91.4 (374)**	80.7 (25) *	92.0 (367)	92.3 (12)
Simple sugar (<5% kcal/day)	47.3 (382)	34.1 (15)	44.3 (181)	32.3 (10)	50.4 (201)	38.5 (5)
MUFA/SFA ratio (≥2:1)	0.37 (3)	2.27 (1)	**0.24 (1)**	3.23 (1) *	0.50 (2)	0.00 (0)
Energy intake from breakfast (20–25% kcal/day)	21.0 (170)	20.5 (9)	21.8 (89)	29.0 (9)	20.3 (81)	0.0 (0)

BQI: Breakfast Quality Index; X: mean; SD: standard deviation; PRI: population reference intakes; MUFA: monounsaturated fatty acids; SFA: saturated fatty acids. For comparison of the BQI, the Mann–Whitney U test is used. For comparison of the items, the χ² test is used. Differences according to the HOMA-IR are indicated with an asterisk and bold type (* *p* < 0.05).

**Table 4:** Associations between the BQI and its components, and IR in the whole sample, according to sex. Logistic regression analysis.

	Total (n = 852)		Girls (n = 440)		Boys (n = 412)	
	Model 1 OR 95% CI	Model 2 OR 95% CI	Model 1 OR 95% CI	Model 2 OR 95% CI	Model 1 OR 95% CI	Model 2 OR 95% CI
**BQI**						
T1 (BQI < 4 points)	1	1	1	1	1	1
T2 (BQI = 4 points)	0.965 (0.427–2.184)	0.995 (0.408–2.427)	1.911 (0.528–6.915)	1.855 (0.491–7.015)	0.473 (0.148–1.513)	0.478 (0.128–1.780)
T3 (BQI > 4 points)	0.856 (0.373–1.964)	0.843 (0.338–2.098)	1.994 (0.560–7.100)	2.094 (0.565–7.761)	0.172 (0.034–0.872) *	0.104 (0.014–0.772) *
**Item BQI**						
Fruits and/or juice consumption	1.876 (1.013–3.471) *	2.203 (1.124–4.314) *	2.238 (1.074–4.665) *	3.202 (1.408–7.281) *	0.885 (0.239–3.280)	0.926 (0.235–3.652)
Dairy products consumption (≥0.5 portion)	1.305 (0.394–4.321)	2.249 (0.510–9.917)	1.611 (0.371–6.999)	1.788 (0.387–8.264)	0.981 (0.124–7.775)	2.163 (0.191–24.499)
Cereals and derivate consumption	1.162 (0.621–2.174)	1.296 (0.651–2.579)	0.974 (0.454–2.089)	1.100 (0.480–2.520)	1.378 (0.454–4.185)	1.589 (0.445–5.667)
Fruits. cereals. and dairy product in the breakfast	1.755 (0.844–3.648)	1.838 (0.820–4.120)	1.981 (0.875–4.485)	2.407 (0.971–5.963)	0.639 (0.081–5.033)	0.620 (0.073–5.240)
Include olive oil	1.353 (0.635–2.884)	1.453 (0.653–3.231)	1.544 (0.639–3.731)	1.818 (0.710–4.654)	0.952 (0.206–4.396)	0.882 (0.177–4.408)
Include butter or margarine	1.898 (1.026–3.514) *	1.888 (0.976–3.655)	1.719 (0.817–3.618)	1.659 (0.742–3.712)	2.252 (0.740–6.849)	2.712 (0.823–8.935)
Calcium (>20% PRI)	0.478 (0.205–1.113)	0.584 (0.238–1.434)	0.390 (0.150–1.014)	0.467 (0.168–1.293)	1.046 (0.132–8.306)	1.540 (0.179–13.209)
Simple sugar (<5% kcal/day)	0.577 (0.305–1.092)	0.522 (0.263–1.036)	0.600 (0.276–1.306)	0.537 (0.233–1.236)	0.616 (0.198–1.914)	0.459 (0.131–1.602)
MUFA/SFA ratio (≥2:1)	6.240 (0.636–61.246)	11.702 (0.902–151.810)	13.600 (0.830–222.865)	16.079 (0.872–296.573)	-	-
Energy intake from breakfast (20–25% kcal/day)	0.940 (0.444–1.989)	0.769 (0.339–1.746)	1.475 (0.656–3.318)	1.368 (0.565–3.314)	-	-
**Item—Fruits and/or juice consumption divided**						
Consume whole fruit ^1^	0.831 (0.320–2.155)	0.836 (0.304–2.296)	0.862 (0.291–2.551)	1.153 (0.356–3.731)	0.609 (0.077–4.792)	0.393 (0.046–3.322)
Consume natural juice ^2^	2.232 (1.168–4.267) *	2.572 (1.255–5.271) *	2.713 (1.279–5.754) *	3.147 (1.370–7.231) *	0.917 (0.199–4.235)	1.517 (0.302–7.620)
**Item—Include butter or margarine divided**						
Include butter ^3^	2.296 (1.213–4.346) *	2.387 (1.184–4.812) *	2.297 (1.058–4.990) *	2.170 (0.921–5.109)	2.416 (0.770–7.581)	2.907 (0.843–10.028)
Include Margarine ^4^	0.977 (0.340–2.807)	1.169 (0.386–3.540)	0.572 (0.132–2.480)	0.680 (0.148–3.124)	2.158 (0.458–10.175)	2.762 (0.511–14.920)

Model 1: Crude model. Model 2: Adjusted for age. sex (in total sample), activity coefficient, percentage of fat mass and zBMI. ^1^ Model 2: adjusted for consuming natural juice. ^2^ Model 2: adjusted for consuming whole fruit. ^3^ Model 2: adjusted for including butter. ^4^ Model 2: adjusted for including margarine. BQI: Breakfast Quality Index; PRI: population reference intakes; MUFA: monounsaturated fatty acids; SFA: saturated fatty acids. The risk associated with breakfast energy intake (20–25%) and the MUFA/SFA ratio (≥2:1) could not be calculated in boys, as no boy with IR met the recommendations for these items. Significant differences are marked with asterisks and bold type (* *p* < 0.05).

## Data Availability

The data presented in this study are available from the corresponding author upon request.

## Supplementary Materials

The following supporting information can be downloaded at https://www.mdpi.com/article/10.3390/ijerph20021181/s1, Table S1. Dietary data for breakfast and daily according to sex. Table S2. Anthropometric, biochemical and dietary data for breakfast and daily according to the BQI tertiles. Table S3. Dietary data for breakfast and daily, according to sex and HOMA-IR.
