# Supplementary material for: Breakfast Quality and Insulin Resistance in Spanish Schoolchildren: A Cross-Sectional Study
Source: Int J Environ Res Public Health. 2023 Jan 9;20(2):1181. doi: 10.3390/ijerph20021181 (PMC9859171; doi:10.3390/ijerph20021181)
Supplement: Supplementary file 1 [file ijerph-20-01181-s001.zip › ijerph-2111489-supplementary.pdf]

Table S1. Dietary data for breakfast and daily according to sex.

	**Total (n=852)**	**Girls (n=440)**	**Boys (n=412)**
**Dietary intake at breakfast**			
Fruits and juice(g/day)	29.4±57.3	**33.2±59.4**	**25.4±54.8***
Fruits (g/day)	7.7±28.1	8.5±29.2	6.8±26.9
Juices (g/day)	21.7±50.6	**24.7±53.3**	**18.6±47.3***
Dairy (g/day)	201.6±68.5	199.1±69.2	204.3±67.7
Cereals (g/day)	15.3±18.3	15.9±18.1	14.6±18.5
Olive oil (g/day)	0.56±1.59	0.58±1.66	0.54±1.51
Butter or margarine(g/day)	1.98±4.08	1.98±3.97	1.98±4.20
Butter (g/day)	1.35±3.40	1.36±3.57	1.33±3.22
Margarine (g/day)	0.63±2.57	0.61±2.14	0.65±2.96
Energy intake (kcal)	371±138	362±130	381±147
Energy intake (% kcal/day)	17.7±5.8	17.5±5.8	17.8±5.9
Proteins (% kcal/day)	2.16±0.82	2.18±0.73	2.15±0.90
Lipids (% kcal/day)	6.4±2.9	6.3±2.9	6.6±2.9
SFA (% kcal/day)	3.1±1.3	**3.0±1.3**	**3.2±1.3***
MUFA (% kcal/day)	2.0±1.1	1.9±1.1	2.0±1.1
PUFA (% kcal/day)	0.49±0.41	0.49±0.38	0.49±0.44
Carbohydrates (% kcal/day)	8.9±3.2	9.0±3.2	8.8±3.2
Simple sugar (% kcal/day)	5.5±2.1	5.5±2.1	5.4±2.1
Free sugar (% kcal/day)	2.40±1.46	2.40±1.52	2.40±1.39
Fibre (g/day)	1.87±1.80	1.93±2.13	1.82±1.37
Calcium(mg/day)	322.1±105.5	317.4±106.5	327.1±104.3
**Daily dietary intake**			
HEI-2015 (score) #	59.1±8.5	**60.3±8.5**	**57.9±8.4***
Energy intake (kcal/day)	2105±350	**2066±338**	**2146±358***
Underestimation (%) #	-1.8±22.8	**-10.7±22.8**	**7.8±18.6***
Proteins (% kcal/day)	15.6±2.3	15.6±2.3	15.5±2.3
Lipids (% kcal/day)	41.8±4.8	41.8±4.7	41.8±4.9
SFA (% kcal/day)	14.4±2.1	**14.2±2.1**	**14.6±2.2***
MUFA (% kcal/day) #	16.9±2.7	16.9±2.8	16.9±2.7
PUFA (% kcal/day)	5.9±1.8	**6.0±1.9**	**5.7±1.6***
Carbohydrates (% kcal/day)	41.0±5.1	40.9±5.0	41.1±5.2
Simple sugar (% kcal/day) #	20.4±4.6	20.4±4.5	20.4±4.7
Free sugar (% kcal/day)	8.5±3.6	8.3±3.6	8.7±3.6
Fibre (g/day)	16.3±4.8	16.4±4.8	16.3±4.7
Calcium(mg/day) #	965.0±221.9	**946.6±217.8**	**984.6±224.9***

Data are shown as mean ± standard deviation. MUFA: monounsaturated fatty acids; PUFA: polyunsaturated; SFA: saturated fatty acids. HEI-2015: healthy eating index. Most variables follow a non-parametric distribution; variables with normal distribution were marked (#). For a comparison of means, the Mann–Whitney U-test was used if the distribution of variables was not normal, and Student's t-test for normal distributions. Differences between sexes are indicated by asterisks and bold type (*p<0.05).

Table S2. Anthropometric, biochemical and dietary data for breakfast, and daily according to BQI tertiles.

	**Total**			**Girls**			**Boys**		
	**T1 (n=157)**	**T2 (n=347)**	**T3 (n=348)**	**T1 (n=73)**	**T2 (n=174)**	**T3 (n=193)**	**T1 (n=84)**	**T2 (n=173)**	**T3 (n=155)**
**Anthropometric data**									
BMI (kg/m^2)	18.8±2.8	18.9±3.0	19.0±3.3	18.9±2.8	18.9±2.9	18.9±3.1	18.7±2.7	19.0±3.2	19.2±3.6
zBMI- S	0.63±1.03	0.68±1.12	0.70±1.18#	0.58±1.04	0.56±1.01	0.58±1.08#	0.68±1.03	0.81±1.21	0.86±1.29
Body fat (%)	27.1±5.5	27.7±5.6	27.7±5.8	29.5±4.4	29.5±4.5	29.1±4.9#	25.1±5.5	25.8±6.1	26.1±6.3
**Physical Activity**									
Activity coefficient	1.52±0.11	1.52±0.11	1.54±0.10	1.52±0.12	1.52±0.11	1.52±0.10#	1.53±0.11	**1.52±0.10**	**1.55±0.11b**
**Biochemical data**									
Glucose (mg/dL) – S	83.9±10.1	84.3±10.2	84.8±8.9	81.7±9.5	83.4±11.2	83.9±8.9	85.7±10.3	85.1±9.1	85.8±8.8
Insulin (mcU/mL) – S	6.4±4.7	6.3±4.3	6.2±4.3	6.9±5.5	7.1±4.5	7.1±4.8	5.9±3.9	5.6±3.8	5.2±3.2
HOMA-IR – S	1.35±1.13	1.33±0.93	1.31±0.92	1.45±1.37	1.48±1.00	1.49±1.04	1.27±0.88	1.18±0.84	1.09±0.70
**Dietary intake at breakfast**									
Fruits and juice (g/day) -B	7.9±38.0	**20.5±51.0a**	**48.0±64.6ab**	**11.7±50.2**	**19.6±49.6**	**53.5±64.3ab**	**4.5±22.4**	**21.4±52.5a**	**41.2±64.4ab**
Fruit (g/day) -B	**1.5±11.8**	**4.5±19.7**	**13.7±37.8ab**	**1.6±14.0**	**3.4±14.2**	**15.7±40.1ab**	**1.4±9.4**	**5.5±24.0**	**11.3±34.7ab**
Juices (g/day) -B	**6.4±35.6**	**16.1±47.4**	**34.3±56.2ab**	**10.0±48.6**	**16.2±47.6**	**37.9±57.0ab**	**3.2±17.6**	**15.9±47.3**	**29.9±55.2ab**
Dairy (g/day) -B	**186.5±94.1**	**201.3±61.9**	**208.8±59.6ab**	186.0±94.1	**194.5±62.3**	**208.1±62.8b**	186.9±94.7	208.1±60.8	209.7±55.4
Cereals (g/day) -B	**11.2±20.4**	**9.9±16.4**	**22.5±16.7ab**	**9.7±15.9**	**11.2±17.7**	**22.5±17.2ab**	**12.4±23.6**	**8.6±15.1**	**22.5±16.1ab**
Olive oil(g/day) -B	**0.01±0.13**	**0.08±0.62**	**1.29±2.21ab**	**0.00±0.00**	**0.10±0.69**	**1.23±2.25ab**	**0.02±0.18**	**0.06±0.55**	**1.37±2.15ab**
Butter or margarine (g/day) -B	**3.30±5.53**	**1.74±3.60a**	**1.62±3.63a**	**3.19±4.87**	1.81±3.85	**1.67±3.63a**	**3.40±6.07**	1.67±3.34	**1.56±3.65a**
Butter (g/day)	1.86±4.26	1.30±3.28	1.16±3.06	1.93±4.51	1.26±3.50	1.24±3.23	1.80±4.06	1.35±3.06	1.05±2.85
Margarine (g/day) -B	**1.45±4.22**	**0.43±1.74a**	**0.46±2.19a**	**1.26±2.87**	0.55±1.92	**0.43±1.95a**	1.61±5.12	0.32±1.54	0.51±2.46
Energy intake (kcal/day) -SB	**383±186**	**342±123a**	**394±122ab**	368±168	**326±107**	**392±124b**	397±201	**358±136**	**397±119b**
(% kcal/day) -B	**18.4±7.2**	**16.4±5.4a**	**18.7±5.3b**	17.7±6.9	**16.1±5.2**	**18.7±5.6b**	**19.1±7.5**	**16.6±5.5a**	**18.6±5.0b**
Proteins (% kcal/day) -B	**1.98±0.77**	**2.00±0.63**	**2.41±0.94ab**	**1.99±0.74**	**2.00±0.65**	**2.41±0.74ab**	**1.97±0.80**	**2.00±0.61**	**2.41±1.14ab**
Lipids (% kcal/day) -B	**7.0±3.7**	**5.8±2.6a**	**6.7±2.6b**	**6.7±3.8**	**5.6±2.5a**	**6.7±2.7b**	7.1±3.7	6.1±2.6a	6.8±2.5#
SFA (% kcal/day) #	3.3±1.8	3.0±1.3	3.0±1.2	3.2±1.8	2.8±1.2	3.0±1.2	3.4±1.8	3.1±1.3	3.1±1.1
MUFA (% kcal/day) -B	**2.0±1.3**	**1.7±0.9a**	**2.2±1.1ab**	2.0±1.4	**1.7±0.9**	**2.2±1.2b**	2.0±1.3	**1.8±0.9**	**2.2±1.0b**

	**Total**			**Girls**			**Boys**		
	**T1 (n=157)**	**T2 (n=347)**	**T3 (n=348)**	**T1 (n=73)**	**T2 (n=174)**	**T3 (n=193)**	**T1 (n=84)**	**T2 (n=173)**	**T3 (n=155)**
PUFA (% kcal/day)	0.52±0.50	**0.45±0.39**	**0.51±0.38b**	0.51±0.47	**0.44±0.35**	**0.52±0.36b**	0.52±0.52	**0.45±0.44**	**0.50±0.40b**
Carbohydrates (% kcal/day) -B	**9.4±3.7**	**8.3±3.1a**	**9.3±3.1b**	9.2±3.3	**8.3±3.1**	**9.5±3.2b**	**9.6±3.9**	**8.3±3.1a**	**9.1±2.9b**
Simple sugar (% kcal/day) -B	**5.9±2.3**	**5.1±2.0a**	**5.6±2.2ab**	5.9±1.8	**5.1±2.0a**	**5.7±2.2b**	**5.9±2.6**	**5.1±1.9a**	**5.3±2.1a**
Free sugar (% kcal/day) -B	**3.1±1.5**	**2.3±1.4a**	**2.1±1.4ab**	**3.1±1.5**	**2.4±1.6a**	**2.2±1.4a**	**3.1±1.6**	**2.3±1.2a**	**2.1±1.3a**
Fibre (g/day) -B	**1.61±1.01**	**1.60±1.04**	**2.27±2.48ab**	**1.58±0.91**	**1.54±0.94**	**2.41±2.97ab**	**1.63±1.09**	**1.65±1.13a**	**2.11±1.67b**
Calcium(mg/day) -B	**305.5±137.9**	**316.3±93.9**	**335.4±98.1ab**	**298.1±134.2**	**305.5±95.2**	**335.6±102.1ab**	312.0±141.6	327.1±91.5	335.3±93.1
**Daily dietary intake**									
HEI-2015 (score) # -SB	**57.3±8.8**	**58.1±8.7**	**61.1±7.9ab**	**58.4±8.9**	**59.1±9.0**	**62.2±7.5ab**	**56.4±8.7**	**57.0±8.3**	**59.6±8.1ab**
Energy intake (kcal/day) -SB	**2033±355**	**2115±369a**	**2127±325 # a**	**2004±326**	2057±364	**2098±317a**	**2058±378**	**2173±366a**	2163±332#
Underestimation (%) - S	**2.4±20.4**	-1.6±22.6	**-3.8±23.9a**	-5.9±19.5	-9.8±23.3	-13.2±23.3#	9.7±18.4	6.6±18.6	8.0±18.8#
Proteins (% kcal/day)	**15.3±2.3**	15.5±2.3	**15.8±2.4a**	15.5±2.1	15.5±2.4	15.9±2.3	15.2±2.5	15.4±2.2	15.7±2.4
Lipids (% kcal/day)	42.2±5.4	41.6±4.8	41.9±4.5	42.2±4.6	41.8±5.0	41.7±4.4	42.1±6.0	41.5±4.5	42.0±4.6#
SFA (% kcal/day)	14.4±2.5	14.5±2.1	14.3±2.0#	14.4±2.3	14.2±2.2	14.2±1.9#	14.4±2.6	14.7±2.0	14.5±2.0
MUFA (% kcal/day)	16.9±2.9	16.9±2.8	16.9±2.6#	16.9±2.6	17.0±2.9	16.8±2.7	16.9±3.1	16.8±2.7	17.0±2.6#
PUFA (% kcal/day) -S	5.9±1.7	5.8±1.8	5.9±1.7	6.1±1.8	6.1±1.9	6.0±1.8	5.8±1.7	5.5±1.6	5.8±1.6#
Carbohydrates (% kcal/day)	41.0±5.6	41.3±5.2	40.7±4.7	40.8±4.6	41.1±5.6	40.7±4.4	41.3±6.4	41.5±4.8	40.6±5.0#
Simple sugar (% kcal/day)	20.5±4.8	20.5±4.5	20.3±4.6	20.8±4.7	20.2±4.7	20.3±4.2	20.3±5.0	20.7±4.2	20.2±5.0
Free sugar (% kcal/day) -B	**9.5±3.8**	**8.9±3.6**	**7.8±3.4ab**	**9.3±3.5**	**8.9±3.8**	**7.5±3.4ab**	**9.6±4.0**	**8.9±3.4**	**8.1±3.5ab**
Fibre (g/day) -B	**15.2±4.5**	**16.3±4.7a**	**16.9±4.8ab**	**15.2±4.0**	**16.1±4.7**	**17.1±5.1ab**	**15.2±4.9**	**16.5±4.8a**	**16.6±4.4a**
Calcium(mg/day) -SB	**904±225.9**	**967±224.8a**	**991±212.4a**	**889±227.1**	**930±206.2**	**983±218.8ab**	**917±225.4**	**1.004±236.9a**	**1000±204.5#a**

Data are shown as mean ± standard deviation. T1: <4 points, T2: 4 points and T3: >4 points. MUFA: monounsaturated fatty acids; PUFA: polyunsaturated; SFA: saturated fatty acids. HEI-2015: healthy eating index. Most variables follow a non-parametric distribution; variables with normal distribution were marked (#). For comparison of means, the Mann–Whitney U-test was used if the distribution of variables was not normal, and Student's t-test for normal distributions. Two-way ANOVA analysis: S: differences by sex; B: differences by BQI score. Significant differences are indicated by letters and bold type (a: differences with T1, b: differences with T2, $p<0.05$).

Table S3. Dietary data for breakfast and daily, according to sex and HOMA-IR.

	**Total**		**Girls**		**Boys**	
	**HOMA-IR ≤3.16 (n=808)**	**HOMA-IR >3.16 (n=44)**	**HOMA-IR ≤3.16 (n=409)**	**HOMA-IR >3.16 (n=31)**	**HOMA-IR ≤3.16 (n=399)**	**HOMA-IR >3.16 (n=13)**
**Dietary intake at breakfast**						
Fruits and juice(g/day) – S	29.0±57.4	38.0±56.0	**31.9±59.1**	**49.5±61.6***	25.9±55.4	10.7±24.9
Fruit (g/day)	7.9±28.8	3.4±10.7	8.8±30.1	4.6±12.6	7.1±27.3	0.5±1.7
Juices (g/day) -S	**21.0±50.3**	**34.7±54.3***	**23.1±52.5**	**44.9±60.1***	18.9±47.9	10.3±25.0
Dairy (g/day)	202.2±69.1	190.8±55.4	199.9±69.8	188.4±60.3	204.6±68.4	196.4±43.4
Cereals (g/day)	15.4±18.5	13.9±14.2	16.1±18.4	13.8±14.2	14.6±18.7	14.2±14.8
Olive oil(g/day) -R	0.54±1.49	0.91±2.83	0.53±1.45	1.23±3.32	0.55±1.53	0.16±0.47
Butter or margarine(g/day)	**1.90±3.98**	**3.39±5.49***	1.84±3.72	3.79±6.21	1.96±4.23	2.44±3.16
Butter (g/day) – I	**1.26±3.24**	**2.96±5.44***	**1.20±3.24**	**3.50±6.28***	1.32±3.25	1.67±2.26
Margarine (g/day)	0.64±2.62	0.43±1.38	0.64±2.19	0.28±1.11	0.65±2.99	0.77±1.88
Energy intake (kcal/day)	370±139	380±132	359±129	390±138	382±148	356±118
Energy intake (% kcal/day)	17.6±5.7	19.4±7.3	17.4±5.6	19.6±7.5	17.8±5.8	18.7±7.0
Proteins (% kcal/day)	2.16±0.82	2.22±0.76	2.18±0.73	2.21±0.78	2.14±0.91	2.25±0.73
Lipids (% kcal/day)	6.3±2.8	7.5±3.5	**6.1±2.8**	**7.8±3.8***	6.6±2.9	6.6±2.5
SFA (% kcal/day)	3.0±1.3	3.5±1.6	**2.9±1.3**	**3.6±1.7***	3.2±1.3	3.3±1.4
MUFA (% kcal/day) -R	**1.9±1.1**	**2.4±1.6***	**1.9±1.0**	**2.6±1.8***	2.0±1.1	1.9±0.8
PUFA (% kcal/day)	**0.48±0.41**	**0.58±0.36***	**0.48±0.37**	**0.63±0.39***	0.49±0.45	0.47±0.26
Carbohydrates (% kcal/day)	8.9±3.2	9.3±3.6	8.9±3.2	9.4±3.4	8.8±3.2	9.0±4.1
Simple sugar (% kcal/day)	5.4±2.1	5.7±2.2	5.5±2.1	5.7±2.1	5.4±2.1	5.6±2.6
Free sugar (% kcal/day)	2.4±1.5	2.4±1.5	2.4±1.5	2.3±1.6	2.4±1.4	2.5±1.4
Fibre (g/day) – SR	1.84±1.29	2.55±5.68	1.85±1.20	3.00±6.73	1.83±1.38	1.50±1.00
Calcium(mg/day)	322.7±106.6	312.0±83.6	317.8±107.6	312.7±92.4	327.6±105.4	310.2±61.0
**Daily dietary intake**						
HEI-2015 (score)	59.2±8.5	57.3±8.6#	60.5±8.4	57.8±9.4	57.9±8.5	56.1±6.4#
Energy intake (kcal/day) #	**2110±350**	**2004±334***	2071±337	1998±361	2150±360	2019±270
Underestimation (%) – SI	**-2.1±22.8**	**5.0±22.2***	**-11.4±22.7**	**-1.4±22.0***	**7.4±18.6**	**20.1±14.6# ***

	Total		Girls		Boys	
	**HOMA-IR ≤3.16 (n=808)**	**HOMA-IR >3.16 (n=44)**	**HOMA-IR ≤3.16 (n=409)**	**HOMA-IR >3.16 (n=31)**	**HOMA-IR ≤3.16 (n=399)**	**HOMA-IR >3.16 (n=13)**
Proteins (% kcal/day)	15.6±2.3	15.7±2.3	15.6±2.3	15.6±2.1	15.5±2.3	15.9±2.9
Lipids (% kcal/day) #	41.8±4.8	42.1±5.2	41.8±4.7	42.1±4.4	41.8±4.8	42.1±6.8
SFA (% kcal/day)	14.4±2.1	14.7±2.2#	14.2±2.1	14.7±2.2#	14.6±2.1	14.9±2.5
MUFA (% kcal/day) #	16.9±2.7	17.1±3.0	16.9±2.8	17.2±2.7	16.9±2.7	16.7±3.7
PUFA (% kcal/day)	5.9±1.7	5.5±1.8	**6.1±1.8**	**5.5±1.8**# *	5.7±1.6	5.6±1.8
Carbohydrates (% kcal/day)	41.0±5.1	40.5±4.9#	40.9±5.0	40.6±4.4#	41.2±5.2	40.4±6.3
Simple sugar (% kcal/day)	20.4±4.6	20.4±3.9	20.3±4.5	20.5±3.8	20.4±4.7	20.3±4.4#
Free sugar (% kcal/day)	8.5±3.6	8.4±3.7	8.4±3.6	8.1±3.4	8.7±3.6	9.0±4.4#
Fibre (g/day)	16.3±4.6	17.3±7.4	16.3±4.5	17.3±8.4	16.2±4.7	17.2±4.3
Calcium(mg/day)	964.0±224.3	983.5±174.4	943.9±220.7	981.8±173.0	984.5±226.3	987.4±184.5#

Data are shown as mean ± standard deviation. MUFA: monounsaturated fatty acids; PUFA: polyunsaturated; SFA: saturated fatty acids. HEI-2015: healthy Eating Index. Most variables follow a non-parametric distribution; variables with normal distribution were marked (#). For comparison of means, the Mann–Whitney U-test was used if the distribution of variables was not normal, and Student's t-test for normal distributions. Two-way ANOVA analysis: S: differences according to sex; I: differences according to insulin resistance (IR) score; R: interaction between sex and IR. Differences according to HOMA-IR are indicated with an asterisk and bold type (*p<0.05).
